# Re-programming immunosurveillance in persistent non-infectious ocular inflammation

**DOI:** 10.1016/j.preteyeres.2018.03.001

**Published:** 2018-07

**Authors:** Simon J. Epps, Joanne Boldison, Madeleine L. Stimpson, Tarnjit K. Khera, Philippa J.P. Lait, David A. Copland, Andrew D. Dick, Lindsay B. Nicholson

**Affiliations:** a Academic Unit of Ophthalmology, Bristol Medical School, Faculty of Health Sciences, University of Bristol, BS8 1TD, UK; b Division of Infection and Immunity, Cardiff University School of Medicine, Cardiff, CF14 4XN, UK; c School of Cellular and Molecular Medicine, Faculty of Biomedical Sciences, University of Bristol, BS8 1TD, UK; d UCL-Institute of Ophthalmology and National Institute for Health Research (NIHR) Biomedical Research Centre at Moorfields Eye Hospital and University College London Institute of Ophthalmology, EC1V 2PD, UK

**Keywords:** Uveitis, EAU, Immunosurveillance, Angiogenesis, Ectopic lymphoid tissue, ACAID, Anterior chamber-associated immune deviation, BRB, Blood retinal barrier, CNS, Central nervous system, CSF, Cerebrospinal fluid, EAU, Experimental autoimmune uveitis, ELS, Ectopic lymphoid-like structures, FDC, Follicular dendritic cells, IRBP, Interphotoreceptor retinoid-binding protein, MHC, Major histocompatibility complex, NO, Nitric oxide, OCT, Optical coherence tomography, RPE, retinal pigment epithelium, S1P, Sphingosine-1-phosphate, Tregs, T regulatory cells, VEGF, Vascular endothelial growth factor

## Abstract

Ocular function depends on a high level of anatomical integrity. This is threatened by inflammation, which alters the local tissue over short and long time-scales. Uveitis due to autoimmune disease, especially when it involves the retina, leads to persistent changes in how the eye interacts with the immune system. The normal pattern of immune surveillance, which for immune privileged tissues is limited, is re-programmed. Many cell types, that are not usually present in the eye, become detectable. There are changes in the tissue homeostasis and integrity. In both human disease and mouse models, in the most extreme cases, immunopathological findings consistent with development of ectopic lymphoid-like structures and disrupted angiogenesis accompany severely impaired eye function. Understanding how the ocular environment is shaped by persistent inflammation is crucial to developing novel approaches to treatment.

## 1 Introduction

The immune response addresses an enormous variety of challenges across many different time-scales from acute viral illness, cleared within a week, to chronic infections, like tuberculosis and herpes viruses, that can remain dormant for decades. A key determinant of the outcome is whether the infection can be eradicated: if it cannot, the environment may be remodelled, to keep pathogens in check. Organ systems demonstrate a wide range of tissue specific flexibility in their adaptations to changes in the environment (Hussell and Bell, 2014) and part of this remodelling involves re-programming patterns of immunosurveillance. When the cause of chronic inflammation is not infection but autoimmunity, elimination of the inciting antigen is often incompatible with survival. Instead, in many cases, an uneasy compromise between the tissue and the immune system is evident, perhaps punctuated by fluctuations in the status of the local inflammation that manifests clinically as remissions and relapses.

No organ system is exempt from the development of autoimmunity. Immune privilege shapes the nature and tempo of immunosurveillance, but all tissues submit to the demands of the immune system, to report on their well-being and integrity. When these reports are unsatisfactory, the consequences in terms of tissue damage are frequently profound. And in addition to harm caused by acute inflammation, a previously healthy tissue may be compelled in the long term to submit to much more rigorous scrutiny than is customary.

Accommodating such change in tissues is a necessary requirement for immune memory. The balance of importance between the central mechanisms of immune memory, that provide recirculating effectors which target diseased tissue, and locally based immune responses is difficult to estimate. There is growing evidence that local tissue responses can play a critical role in defending an individual from reinfection at specific sites (Schenkel and Masopust, 2014; Steinert et al., 2015). It is also clear from recent work in animal models and human disease, that the long-term local changes which occur are important contributors to the severity of disease. These effects manifest across a multiplicity of individual core functions including: immune tissue neogenesis, angiogenesis, stromal remodelling and the local expression of co-inhibitory molecules. Whether these represent individual independent modules, or an interlocking programmatic response is not clear. But in different diseases, therapeutically successful targeting of these functions offers a paradigm to intervene, with the goal of resetting the inflammatory state of the tissue.

Autoimmune diseases in the eye capture many aspects of the process in both the clinical setting and in disease models. Models have allowed the dissection of the interplay between important core functions, while the introduction of biologics into the clinic have helped establish the close relationship in the underlying pathology between ocular disease and diseases such as rheumatoid arthritis. Understanding the new patterns of immunosurveillance that are consequent upon autoimmune disease will be important for managing future immunotherapies.

## 2 New niches for immunosurveillance

### 2.1 Immune privilege & privileged niches

Our understanding of immune privilege has evolved over the seventy years since the concept was elaborated in seminal investigations of skin grafts transplanted to different tissues (Medawar, 1948). The original notion of antigen sequestration behind anatomical barriers has been revised, as the structures that provide lymph drainage from privileged sites such as the brain have gradually come to be recognised (Andres et al., 1987; Aspelund et al., 2015; Földi, 1966; Louveau et al., 2015); the presence or absence of choroidal lymph drainage from the healthy eye remains controversial (Chan-Ling et al., 2015; Heindl et al., 2015; Koina et al., 2015; Schrodl et al., 2015; Schroedl et al., 2014), but like the brain, autoimmunity in the eye connects to the rest of the immune system (Alex et al., 2011; Xu et al., 2007).

Studies of human immune cells obtained from the CSF of subjects that do not have CNS inflammation are a window on normal immunosurveillance of immune privileged tissue. They find mainly CD4^+^ T cells of an effector memory phenotype (Engelhardt and Ransohoff, 2005; Kivisäkk et al., 2003); the reactivation of occult infections that can arise in patients following blockade of immunosurveillance attests to its importance in sustaining health (Wraith and Nicholson, 2012).

The special immunological status of the eye has been recognised and exploited by many investigators over a number of years. This has produced a large literature which has been extensively reviewed (Niederkorn, 2006; Taylor, 2009). One striking feature is the ability of the eye to respond to the inoculation of foreign cells with a systemic immune deviation that suppresses delayed type hypersensitivity responses. This phenomenon is known as anterior chamber-associated immune deviation (ACAID). Inoculation into the vitreous cavity has similar effects and this phenomenon is known as vitreous cavity-associated immune deviation (Sonoda et al., 2005).

It is clear from animal models that ACAID depends on autonomic innervation and leukocyte recirculation outside the eye for its induction; it is thought to limit bystander damage to from potentially injurious activated immune cells (Lin et al., 2005; Niederkorn, 2006), therefore having a physiological role in sustaining tissue integrity.

**Fig. 1:**
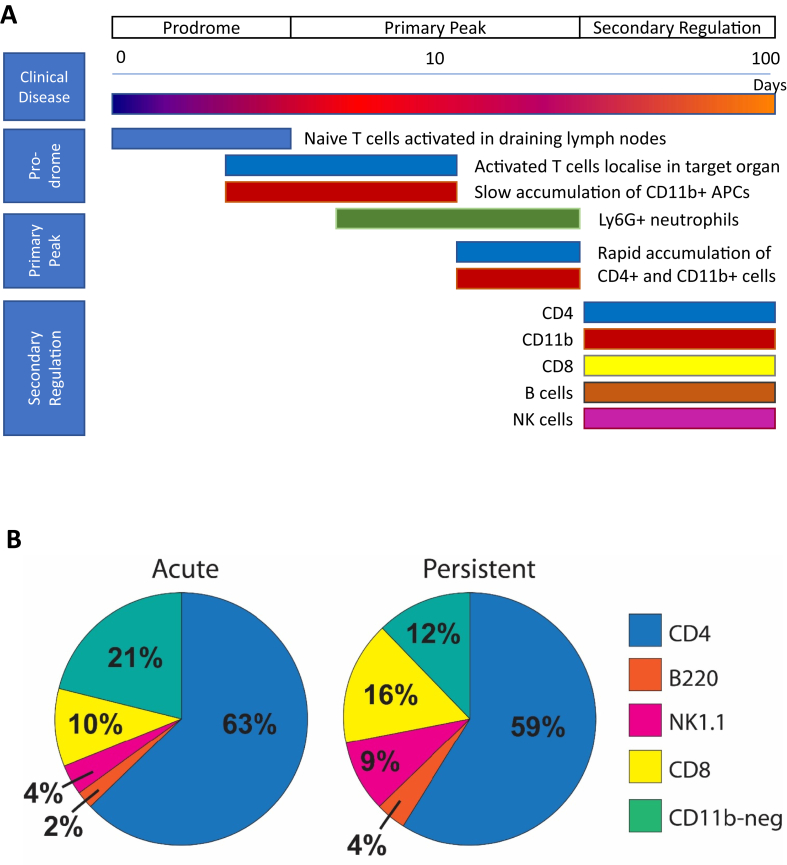
Ocular infiltration by lymphocytes is comprised of a diverse population of cells. (A) The prodrome is characterised by an initial slow localisation of cells to the eye, followed by rapid accumulation and secondary regulation. (B) Lymphocytes were prepared from the vitreous, retina and ciliary tissue of C57BL/6 animals with acute EAU (day 23–25) and persistent disease (day 38–43). Populations of live CD45 positive, CD11b negative cells were quantified by multiparameter flow cytometry. Adapted from Kerr et al., 2008a, Kerr et al., 2008b Prog. Retin. Eye Res. 27, 527–535 & Boldison et al. (2014). J Immunol 192, 4541–4550.

A more pressing concern of this monograph is whether and how processes of immunosurveillance are modified once local inflammation becomes established. Brain and eye tissues, though they are isolated from the circulation by anatomical and functional barriers in health, undergo profound local changes that are triggered by immune responses within the affected tissue (Bechmann et al., 2007; Kerr et al., 2008a; Zhou et al., 2012). In the context of models of ocular organ specific autoimmunity, e.g experimental autoimmune uveitis (EAU), it is feasible to demonstrate that this all plays out in a time frame that can usefully be divided into different phases. Disease is clinically silent following initiation, for example by immunisation with autoantigen, and although sensitive analysis in this phase demonstrates an increase in leukocyte retention in the retina, this is antigen non-specific (Prendergast et al., 1998) (Lait; unpublished data). Clinical disease follows this prodrome, characterised by a large influx of leukocytes that rapidly reach a peak and then partially resolve. Subsequently there is an indefinite period of secondary regulation; in murine experimental models the retina never returns to normal health (Chen et al., 2012; Copland et al., 2008; Kerr et al., 2008a, 2008b; Wang et al., 2011). Persistent inflammation is associated with retinal dysfunction and with the development of niches that favour the retention immune cells in the vicinity of the affected tissue. Fig. 1 and Table 1 summarise the cell content of these different phases, elaborating on how an increasingly rich and diverse set of immune cells can be obtained from the eye as EAU develops.

**Table 1:** Leukocyte content at different phases of EAU.

Cell population	Mouse strain	Normal*	Prodrome**	Peak	Secondary regulation***
CD11b^+^ cells	B10.RIII	660 ± 50	1050 ± 100	104,000 ± 26,800	4980 ± 2110
	C57BL/6	700 ± 30	1400 ± 130	21,730 ± 3880	18,640 ± 4250
CD4^+^ lymphocytes	B10.RIII	25 ± 5	120 ± 15	23,500 ± 5700	3870 ± 1230
	C57BL/6	20 ± 2	215 ± 20	15,340 ± 2950	17,290 ± 3500
CD8^+^ lymphocytes	C57BL/6			2320 ± 440	4640 ± 810

Cells were prepared from the vitreous, retina and ciliary body from normal animals or animals with EAU and quantified as described (Boldison et al., 2014; Kerr et al., 2008b). *Mean ± standard error. Data from 17 or more eyes except B10.RIII peak disease from 7 eyes. **Data pooled from days 5–7; ***Data pooled from days 34–44. Data from (Boldison et al., 2014; Kerr et al., 2008b) and J. Boldison & P. Lait unpublished.

The concept of niches that support cell survival developed from ecology and was first applied by cell biologists to hematopoietic stem cells. A prescient paper published in 1978 (Schofield, 1978) proposed that the basic characteristic of these niches would be a defined anatomy, containing stem cells that could be sustained and reproduce, but which was constrained and therefore limited the maximum stem cell population size (Papayannopoulou and Scadden, 2008). The extension of this framework to lymphocytes produced an elegant approach to analysing their survival and homeostasis, emphasised that different populations (for example naive versus memory cells) interact with specific and different niches, and highlighted competition for resources as a key element of this process (Butcher and Picker, 1996; Freitas and Rocha, 2000). This work was primarily concerned with lymphocytes in the circulation and in secondary lymphoid tissue. The description and definition of the resources that maintain memory cells continues to play an important role in our developing understanding of lymphocyte homeostasis (Sprent and Surh, 2011; Surh and Sprent, 2008). But advances in this area also demonstrated that for certain cell types, the set point of homeostasis can be changed. Experiments quantifying the population sizes of CD8^+^ memory cells (Vezys et al., 2009), showed that the immune system has the capacity to re-program, increase niche capacity, and change in response to infection.

Chronic inflammation of many kinds leads to profound changes in the local anatomy and immune response. This has been described in numerous tissues affected by autoimmunity. In the synovium of patients with rheumatoid arthritis, blood vessels develop lining cells, typical of lymph node high endothelial venules (Jalkanen et al., 1986), that are associated with the accumulation of helper-inducer T cells (Pitzalis et al., 1988). The discovery of cytokines and chemokines that co-ordinate this process, beginning with lymphotoxin (Cyster, 2014; Kratz et al., 1996) led to the concept that the most extreme manifestation of this process was lymphoid neogenesis: a recapitulation outside the immune system of the development of structures associated with lymph nodes (Drayton et al., 2006). Applying these principles to immune privileged tissues such as the eye and brain was initially controversial, but there is now a strong case that lymphoid follicles can develop in the in the cerebral meninges of the central nervous system (CNS). In a study of post-mortem brain tissue of patients with secondary progressive multiple sclerosis, the follicles were found distributed throughout the forebrain, in the meninges entering the cerebral sulci (Magliozzi et al., 2007; Serafini et al., 2004). This was associated with severe cortical demyelination in adjacent tissue and also with poor clinical outcome (Howell et al., 2011). Follicles have also been shown to develop in animal models of MS, where this process is driven by Th17 cells (Peters et al., 2011). Here cells were reported to be in the spinal cord sub-arachnoid space. Therefore, immune privilege is no bar to immune system tissue re-programming, although it may modify its manifestations.

**Fig. 2:**
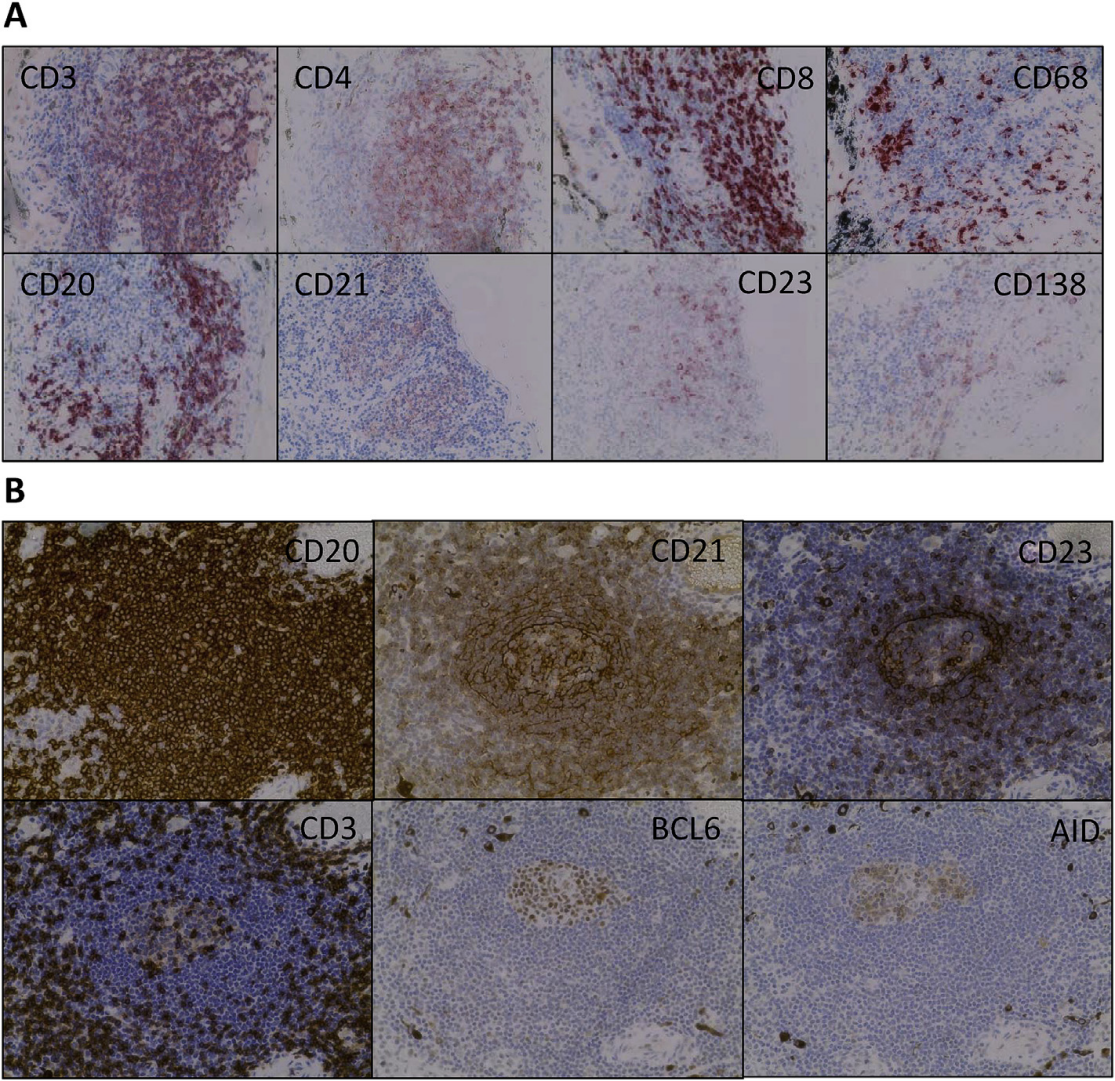
Lymphocyte populations in human uveitis. Lesions stained with different markers of leukocyte phenotype are shown. (A) Immunohistochemistry of the choroid of a 29 year old woman with persistent non-infectious uveitis (Idiopathic uveitis) demonstrating different lymphocyte populations. (B) Serial section of a single lesion showing follicle like structure in human uveitis. Immunohistochemistry images of the choroid of a 28 year old woman with persistent non-infectious uveitis (scleritis, episcleritis and uveitis) demonstrating features of ectopic lymphoid-like structures: T cells surrounding B cells and evidence of active recombination (BCL6+ and AID + cells.) In both cases enucleation was carried out a for blind painful eye, late in the disease process. Photomicrographs show T cells (CD3^+^, CD4^+^ and CD8^+^), B cells (CD20^+^), Follicular B cells(CD23^+^), Plasma cells (CD138), Follicular Dendritic Cells (CD21^+^) and macrophage/monocytes (CD68^+^). Granulomas were not identified in these specimens. All images ×400 magnification.

The chronic inflammatory process in the retina resembles that in the CNS, in that it occurs behind the blood retinal barrier (BRB) in an immune privileged environment. Non-infectious human uveitis presents as a heterogeneous group of diseases, some of which have an underlying autoimmune aetiology (Caspi, 2010; Forrester et al., 2013; Lee et al., 2014). Its animal model, experimental autoimmune uveitis (EAU) correlates well with many of the pathological features that are seen in these human diseases (Copland et al., 2008). T cell receptor (TCR) transgenesis, with retinal autoantigen specific TCR can drive lymphoid neogenesis in the mouse retina (Kielczewski et al., 2016), while in the human eye, tissue obtained from individuals with a history of persistent uveitis, follicles are seen in about 20% of cases (Fig. 2; S. Epps et al. unpublished data).

In the animal model, disease is most often induced by active immunisation using retinal specific antigens and adjuvants, or by the transfer of antigen specific T cells, though other models have also been used (Caspi, 2010; Kerr et al., 2008a; Zhou et al., 2012). The common use of adjuvants in these models may lead to concurrent para-inflammatory reactions to infections components (Forrester et al., 2013; Xu et al., 2009). Studies of EAU often focus on the acute phase of the response, but although EAU has been described as monophasic, sensitive analyses of the disease in the mouse has demonstrated that while the tempo of inflammation it is less after the primary peak of inflammation it remains a dynamic process, furthermore the immune content and immunosurveillance of the tissue is radically altered by the disease (Kerr et al., 2008b) and subsequent studies have confirmed and extended this observation (Chen et al., 2012; Chu et al., 2013; Oh et al., 2011). How these long-term changes over months in mice relate to chronic burnt-out disease in humans requires further exploration.

Because the normal eye contains few lymphocytes, quantifying cell content as EAU develops following immunisation can be used to identify the disease prodrome. This is followed by an exponential increase in the leukocyte content of the eye, which defines a primary peak of disease, and elevated immune cell numbers continue to be measurable through an extended period of secondary regulation (Fig. 1 and Table 1). Differences are observed between mouse strains, with B10.RIII mice recruiting more cells to the eye at peak disease but having a lower cell content during secondary regulation; the opposite pattern is seen in the C57BL/6 strain (Table 1). In the majority of animals, disease status assessed by clinical examination or cell content is comparable in both eyes, but in a significant minority (about 18%), disease may be discordant between individual eyes for an extended period of time (J. Boldison, T Khera unpublished data). This demonstrates a degree of local tissue independence in how pathology manifests, highlighting that the immune response incorporates a negotiation between systemic immunosurveillance and local immune regulation. This may also inform our findings in human disease that follicle maturation occurs with different kinetics in the same eye (see below).

In C57BL/6 animals the dynamics of CD4^+^ T cell recruitment, which peak early, is different from that of CD8^+^ T cells (and B220 positive and NK 1.1 positive cells) which accumulate during secondary regulation between 3 and 6 weeks after the induction of uveitis (Boldison et al., 2014), reaching higher cell densities than are seen at the peak of disease, densities that are maintained for an extended period (at least until day 120 after disease induction - J. Boldison, M. Stimpson unpublished data). This indicates continuous shaping of the local microenvironment as the disease progresses. At later time points (days 60–90) C57BL/6 mice also develop retinal neovascular membranes as a result of a persistent angiogenic process (Chen et al., 2012). These findings emphasise the evolving nature of persistent inflammation in EAU and imply that one result of local autoimmunity is a change in the capacity of the immune privileged tissue to retain populations of immune cells; the tissue has developed new niches that immune cells can inhabit.

### 2.2 Impact on retinal function

Uveitis is a significant burden on retinal tissue and glia-neuron interactions play a key role in health and disease (Vecino et al., 2016). Massive gliosis is one non-specific response, that is induced by many different ocular insults, including persistent inflammation (Nork et al., 1986; Yanoff et al., 1971). It has long been recognised in experimental uveitis in both rodents and primates (Chan et al., 1991; Fujino et al., 1992). Müller cells can inform immune cell function, and in some circumstances glial cells may be profoundly immunosuppressive (Caspi et al., 1987). Many of the responses that are seen in gliosis are stereotypical alterations, that are independent of cause (Bringmann et al., 2006), but glial cells both produce and respond to inflammatory cytokines of innate and adaptive immune response origin (Bringmann et al., 2009). Müller cells are a source of the proinflammatory cytokine IL-8, that promotes neutrophil recruitment to sites of inflammation (Bringmann et al., 2009). Neutrophils are also an important component of the inflammatory infiltrate early in the course of EAU (Kerr et al., 2008b). In late stages of gliosis, Müller cell processes can form glial scars in the sub-retinal space, that inhibit photoreceptor regeneration. This undesirable outcome highlights the dual nature of Müller cells. Essential for neuronal health, but capable of driving irreversible anatomical changes (Bringmann et al., 2006). A key component of this process is the presence of activated microglia, which produce neurotrophic factors that modulate Müller cells and modify their support of photoreceptors, influencing the balance between regeneration and apoptosis (Harada et al., 2002). Isolated astrocytes are themselves also able to activate uveitogenic T cells (Jiang et al., 2008) and there is some evidence that they may respond to the cytokine microenvironment differently than RPE cells (Ke et al., 2009). It is clear that cells originating in the local environment can interact with accumulating immune cells.

More subtle examples of reorganisation are recognised when inflammation is less marked but not completely resolved. Under these circumstance there may be movement of the microglia and recruited macrophages to the subretinal space, where they are retained rather than being cleared by normal homeostatic processes (Guillonneau et al., 2017). These changes in location are sometimes associated with changes in function, for example upregulation of the phagocytic and antigen processing capability of these cells. By such changes the ability of the tissue to interact with the adaptive immune system can be subtly enhanced, altering the likelihood of full activation.

### 2.3 Local scale and scope

One explanation for the reorganisation of immune cells in immune privileged tissues is that this allows a local response tailored to stimuli within the tissue. It raises questions about what these signals are and how autonomous they might be. Regulation of immune cell retention in tissues depends on the integration of signals from cell surface receptors that interact with soluble and cell surface ligands. These signals modulate many different aspects of cell function including activation state, location, motility, metabolism and differentiation. But are the molecular cues that regulate these processes local or systemic in nature? Should we envision immune reorganisation as arising in response to global instruction, or is a better model a series of loosely co-ordinated independent expressions of antigen specific immunity?

The shortest distance over which effector functions act is between one cell and its neighbour. The exquisite specificity that can be achieved has been nicely demonstrated for CD8^+^ T cell killing, where within a skin graft composed of a mixture of MHC haplotypes, only those cells that elicit an allogeneic immune response are killed while non-allogenic neighbours survive (Rosenberg and Singer, 1988). These cell-cell contact dependent events rely on the focused release of cytotoxic molecules.

Studies directed at determining the maximum distance over which cytokines elicit effector functions have demonstrated that they can work over quite different scales. Some investigators have highlighted the importance of directional secretion of cytokines into the synaptic cleft (Huse et al., 2006; Sabatos et al., 2008), while others have provided evidence that cytokines determine cell fate throughout a whole lymph node (Olekhnovitch et al., 2014; Perona-Wright et al., 2010).

Soluble effector molecules can co-ordinate acute tissue responses in other ways. The range over which signalling by interferon-gamma (IFNγ) to macrophages establishes a killing zone is more than 80 μm from the activated T cells (Müller et al., 2012). Then the high levels of nitric oxide (NO), produced in response to IFNγ signalling stimulated by intracellular Leishmania infection, entrains the elimination of parasites whether or not the individual macrophages have the capacity themselves to produce NO. This was shown by studying mixtures of cells competent or not competent for NO production: As enough NO becomes available throughout the tissue the individual phenotype of the macrophage is irrelevant to pathogen destruction (Olekhnovitch et al., 2014). NO is also seen to be acting at a distance in EAU, and as well as activating macrophage/microglia, it is one of a number of effector molecules that limit the local proliferation of T cells (Raveney et al., 2009, 2010).

How soluble molecules have action at a distance, called ‘scaling effects’, have been studied in other areas of cell biology. In development, ‘source-sink’ models have been explored since the 1970s to explain the generation of morphogen gradients (Rogers and Schier, 2011). A recent report applying these concepts to cytokines such as IL-2 illuminate the application of this principle in the immune system, focusing on the balance between diffusion and consumption. In these experiments, the density of cytokine receptor bearing cells, which can remove cytokines from the environment, determines the distance over which the cytokines act (Oyler-Yaniv et al., 2017). This work highlights how dynamic microenvironments can be, with their size changing in relation to the amount of cytokine and the density of cytokine consuming cells. This volume can be modelled as a sphere with a radius that varied from 30 to 150 μm. Interestingly, this would produce a local microenvironment on a similar scale to those found in follicles measured in human tonsils (Crocker et al., 1983; Gorfien et al., 1999). But smaller niches have also been described, for example, stem cell niches in *Drosophila* which have recently been reported to have a radius of 25 μm (Liang et al., 2017).

### 2.4 Antigen driven Re-programming

The observation that leukocytes can be obtained from the retina of animals following acute EAU in numbers that far exceed those obtained from healthy animals, or from animals immunized with non-ocular antigens, attests to the changes that the disease process imprints on the local tissue (Table 1). The organisation of these cells in animals and humans varies enormously, from diffusely scattered infiltration, through perivascular accumulations, to structures that resemble organised lymphoid follicles (Chu et al., 2016; Kielczewski et al., 2016; Kleinwort et al., 2016; Murray et al., 1990). The most likely principle driver of this is ongoing antigen presentation. What is the evidence that autoantigens are still present in late disease?

It is known that in EAU there is strain and species associated variability in the degree of photoreceptor destruction (see for example (Chen et al., 2012; Oh et al., 2011)) and in human studies very little retinal tissue may be apparent in end stage disease. Likewise, in some rodent models, complete destruction of the retina has been reported. On the other hand, in the C57BL/6 model of EAU, photoreceptors are preserved at least as late as 120 days after immunisation (Chen et al., 2012). In diseases such as type I diabetes, where it had long been believed that pancreatic beta-cell destruction is complete, this view has been revised. In sensitive evaluations of insulin C-peptide production, evidence has been found for ongoing cell regeneration, long after the onset of clinical disease (Wang et al., 2012). Complete destruction of a target tissue to a level where there is no autoantigen presentation is therefore less common than has been appreciated, and frustrated attempts at regeneration may be a long-term source of autoantigen (Casciola-Rosen et al., 2005).

Immunoregulation also serves to preserve the source of autoantigens. In both infection and autoimmunity, despite the continued presence of antigen, the immune response has capacity to down-regulate local tissue inflammation and target tissue destruction. The literature identifies a number of different mechanisms including the development of tissue specific T regulatory cells (Tregs) (Rosenblum et al., 2011) and the presence of antigen presenting cells whose ability to initiate T cell activation is constrained (Nicholson et al., 2009; Raveney et al., 2010). In keeping with these observations, in models of persistent infection, for example herpes simplex viral infection of the trigeminal nerve, the local response to infected cells is exquisitely balanced between dominant and sub-dominant CD8^+^ T cell populations and between active and sub-clinical inflammation in both mice and humans (St Leger et al., 2013; St. Leger et al., 2011; Verjans et al., 2007).

The multiparameter analyses that have facilitated more comprehensive quantification of recruited cell populations in studies of ocular autoimmunity, have revealed complexity in both immune cell type and cell dynamics in the affected tissue. Many different lymphocytes can be detected (Fig. 1), some of which have relatively short tissue half-lives, some of which are resident in tissue for much longer (Boldison et al., 2014). It is to be anticipated that during secondary regulation this variety of cell phenotypes have a broad range of different functions, but one organising observation, based on the expression of different immune relevant coinhibitory-receptors, and the accumulation of Tregs, is a shift from a tissue tolerating immune activation to one resisting it.

The substantial difference in immune cell recovery from the eyes of animals after they have developed clinical EAU, compared with age matched naive controls, argues strongly against the proposition that during secondary regulation, the tissue returns to a state that is equivalent to that pre-disease. Different lymphocyte populations show distinct and different patterns of accumulation and dynamics in terms of recirculation. Furthermore the retina is not the only compartment altered by the disease process, as in EAU the bone marrow has been identified as a source of CD4^+^ T cells that mediate chronic uveitis (Oh et al., 2011).

### 2.5 Changes in matrix and supporting cells

Of the many cell types involved in tissue remodelling, the least is known about changes in the supporting stromal tissue that contribute to the niches that the infiltrating immune cells occupy. There are significant changes in cell matrix is some models of uveitis. In equine recurrent uveitis, levels of fibronectin and osteopontin in the vitreous fall, and this is accompanied by deposition of fibronectin deeper in the retina, and the almost complete disappearance of osteopontin (Deeg et al., 2011). Data is scarce on stromal cells in the matrix in persistent uveitis, although exogenously derived mesenchymal stem/stromal cells have been reported to protect from EAU by a CCL2-dependent recruitment of myeloid-derived suppressor cells (Lee et al., 2015).

Retinal pigment epithelium (RPE) cells have been investigated extensively. There is good evidence for them playing an important role in sustaining ocular privilege (Ferguson and Griffith, 2006; Stein-Streilein, 2013; Sugita, 2009; Sugita and Streilein, 2003). Many mediators that play a role in this process have been described; some are soluble factors, while others are contact dependent. RPE cells can activate and reprogram lymphocytes, driving the development of Tregs (Sugita et al., 2006, 2008) and macrophages, inducing cells resembling myeloid derived suppressor cells (Tu et al., 2012). These important findings have also been translated to clinical studies (Mandai et al., 2017; Sugita et al., 2016).

The role of the RPE in the face of persistent inflammation is not as clear. In models of AMD, modulation of autophagy can have effects on inflammasome activation (Liu et al., 2016). Culture of RPE cells with inflammatory mediators such as interferon-gamma does not abrogate their ability to suppress immune responses (Sugita, 2009), but this ability is clearly insufficient to prevent the development of leukocyte infiltration and disease in EAU. Although the expression of Fas ligand is a prominent feature of eye tissue, deficiencies in this cell death inducing pathway does not cause spontaneous uveitis. Instead, the absence of this control mechanism exacerbates disease that is induced by conventional means (Ferguson and Griffith, 2006). RPE cells produce factors such as IL-33, that modulate uveitis through the disease course (Barbour et al., 2014; Theodoropoulou et al., 2017), but it has also been reported that the production of inflammatory stress can damage the RPE by mechanisms that depend on IL-6 and IL-8 signalling (Leung et al., 2009; Planck et al., 1992), perhaps rendering them less effective functionally.

IL-6 has been identified as an important inflammatory cytokine in a number of different inflammatory eye diseases (Ohta et al., 2000). In endotoxin induced inflammation IL-6 antagonises the effects of TGF-beta (Ohta et al., 2000), and blocking IL-6 suppresses EAU (Haruta et al., 2011). This work has led to studies of IL-6 blockade in patients.

## 3 Cells and structures

### 3.1 CD4^+^ T cells

EAU is known to be CD4^+^ T cell dependent, because transfer of activated retinal autoantigen specific CD4^+^ T cells into a naive host is sufficient to induce disease (Caspi, 2010). The evolution of pathology depends on these cells crossing the BRB which both in rats (Prendergast et al., 1998) and mice (Xu et al., 2003) is facilitated by T cell activation and can occur independent of antigen specificity. In different EAU models both Th1 and Th17 cells have been shown to cause disease (Luger et al., 2008). Following immunisation with retinal antigens, the accumulation of small numbers of CD4^+^ T cells in the retina is detectable in the preclinical phase of disease, although their precise anatomical site is not well defined (Kerr et al., 2008b). CD11b^+^ monocytes and smaller numbers of Ly-6G^+^ neutrophils also accumulate with kinetics that are indistinguishable from the CD4^+^ cells. The techniques used to identify these cells (flow cytometry of disaggregated tissue) do not distinguish between cells arrested in the tissue bound to blood vessel endothelium and cells deeper within tissues. In lung tissue it has been demonstrated that perfusion does not remove these cells from the tissue (Anderson et al., 2012), so the precise location of cells identified following tissue disaggregation remains an open question.

The relative frequencies of different cell populations that contribute to disease is regulated by the cytokine and chemokine milieu. Early in disease, it is changes in the BRB and the local expression of a number of different chemokines and their receptors that enable the entry of cells into the tissue (Crane and Liversidge, 2008), while the expression of CD62L and CD44 also play a critical role in directing blood monocyte trafficking (Xu et al., 2008). In animals deficient in IFNγ, disease still occurs but there is an increase in the neutrophil content of the infiltrating population compared with monocytes, which is associated with more profound tissue damage (Su et al., 2007). In a transgenic model where antigen was injected directly into the eye, CXCR4 (but not CXCR7) was critical for ocular leukocyte trafficking leading to acute uveitis (Zhang et al., 2009). Less is known about the mechanisms that retain cells within the retina in the long term.

Chronic inflammation in other tissues such as the skin and lung have been characterised more extensively and these findings may be relevant to chronic autoimmune inflammation in the eye. In models of delayed type hypersensitivity, after cells have accumulated, the chemokine receptor CCR7 plays an essential role in the early egress of lymphocytes from tissue (Bromley et al., 2005). Later in the course of inflammation CCR7 independent exit mechanisms also come in to play (Brown et al., 2010). One pathway which has been characterised is the regulation of the sphingosine-1-phosphate (S1P) receptor 1 by CD69. The upregulation of CD69 inhibits cells’ ability to sense gradients of S1P that provide cues for directed exit from a tissue. Other less well characterised mechanisms can be inhibited by pertussis toxin, therefore likely involving G_αi_-coupled receptor signalling. These changes in the mechanisms by which CD4^+^ T cells are retained within niches in tissues are accompanied by alterations in the rate at which lymphocytes traffic out of tissues. In sheep and mice, when inflammation is chronic, there is an increase in the rate at which cells exit tissues (Brown et al., 2010). The important relevance of these studies to uveitis (Boldison et al., 2014; Chen et al., 2012; Copland et al., 2012; Oh et al., 2011; Raveney et al., 2008) is that re-programming under chronic inflammation remains a dynamic process at late stages of disease, with recirculation of CD4^+^ cells, changes in the composition of infiltrating immune cells and changes in their rates of recirculation.

### 3.2 CD8^+^ T cells

Antigen specific cytotoxic CD8^+^ T cells are a critical part of the immune response to many intra-cytoplasmic pathogens, especially viruses. In the mouse, viral infections provoke the expansion of responder cells but also the generation of memory cells. Populations of these memory cells are distributed to different organs where they become long-lived tissue resident cells (Klonowski et al., 2004; Sheridan and Lefrancois, 2011). The distribution to different organs is not uniform, and immune privileged tissues are resistant to the accumulation of memory cells following systemic infection, although they are an important component of the response to local infection (Schenkel and Masopust, 2014; Wakim et al., 2010, 2012). For example, CD4^+^ T cell help regulates the mobilization of CD8^+^ T cells to the vaginal cavity (Nakanishi et al., 2009) and detailed quantification of tissue resident CD8^+^ T cells demonstrated regionalised immunosurveillance (Steinert et al., 2015). CD8^+^ T cells are recognised to play a role in many different autoimmune diseases, including diabetes and diseases that affect the central nervous system (Goverman, 2009; Santamaria, 2010).

Retinal antigen specific CD8^+^ T cells obtained from the periphery of animals with uveitis have been studied and both pathogenic and regulatory roles have been reported. They play an important role in ACAID where they act as efferent inhibitors of uveitis, able to restrict disease even when it has become established (Hsu et al., 2014; Keino et al., 2006; Lin et al., 2005; Peng et al., 2006, 2007; Shao et al., 2005).

CD8^+^ T cells that are obtained from the retina are phenotypically heterogeneous (Boldison et al., 2014). Unlike CD4^+^ T cells which achieve their highest density of infiltration early in EAU, CD8^+^ T cells continue to accumulate as the disease progresses; the number of CD8^+^ T cells that are recovered from retinas in late disease varies with strain and is greater in C57BL/6 animals compared with B10.RIII ((Boldison et al., 2014) and unpublished data). In the C57BL/6 mouse CD8^+^ T cells numbers double between day 21 (peak) and day 42 (secondary regulation). Depleting CD8^+^ T cells before immunisation did not change the course of EAU in rats (Calder et al., 1993) but peripheral depletion at late stages of disease in mice clears these cells from the retina and leads to an increase in cellular infiltration by CD4^+^ T cells and CD11b^+^ macrophages, implying that the presence of CD8^+^ T cells limited the accumulation of other leukocytes (Boldison et al., 2014). The molecular mechanisms that regulate the process of counter regulation are yet to be understood.

Phenotyping CD8^+^ T cells in the retina by flow cytometry during inflammation reveals that most (>90%) have characteristics of effector memory (CD44^hi^CD62L^low^). As disease progresses about half of the CD8^+^ T cells show an increased expression of the co-inhibitory receptor PD-1, which is a key signature of the exhaustion program of CD8^+^ T cells (Wherry, 2011). The part that this inhibitory costimulatory pathway plays in EAU is currently uncertain. Treatment of mice and humans with reagents that block its action are known to exacerbate clinical disease, (J. Boldison and A. Dick personal communication), and this is likely to be an important practical consideration when targeting co-inhibitory receptors for the treatment of human tumours (Antoun et al., 2016).

Because a substantial fraction of CD8^+^ T cells do not express PD-1, this raises the possibility of diversity of function of the CD8^+^ T cells in the microenvironment of the intracellular infiltrate, perhaps relating to the antigen specificity of the cells. In the C57BL/6 strain, we do not detect the expression of Foxp3 or IL-10 expression by CD8^+^ T cells, arguing against an important role for STAT3 modulated IL-10 producing CD8^+^ T cells in this model of disease ((Yu et al., 2013) and J. Boldison unpublished data).

The relevance of CD8^+^ phenotype to human ocular disease goes beyond the role that it plays in the ocular environment. Studies of CD8^+^ T cells obtained from peripheral blood have shown that interrogating these cells can be used as part of a novel diagnostic strategy to distinguish sarcoidosis from Behcet's disease (Candia et al., 2013) although the relationship between tissue resident and circulating CD8^+^ T cells remains to be elucidated. CD8^+^ T cell phenotype is also an important prognostic consideration in other autoimmune diseases (McKinney et al., 2010, 2013, 2015).

The signals that define the niche that CD8^+^ T cells occupy in autoimmune disease are not as well characterised as those that regulate tissue accumulation following infection. One feature of these antigen specific tissue resident memory CD8^+^ T cells that accumulate following the clearance of an acute viral infection is the upregulation of the integrin CD103. In EAU, CD103 is not upregulated, yet memory CD8^+^ T cells are retained in the tissue. As for CD4^+^ T cells, another candidate for the molecular regulation of cell exit is CD69. CD8^+^ T cells in the tissue express high levels of CD69 which limits lymphocytes’ ability to use signals from S1P gradients to regulate trafficking into the circulation. Therefore, the niche could be defined by an inability to escape, rather than by an invitation to stay.

### 3.3 B cells and ectopic lymphoid-like structures

The role of B cells in non-infectious uveitis has not been as extensively studied as that of the T cell or myeloid cell (either in experimental uveitis or human uveitis) although interest in the B cell with respect to uveitis is increasing (Smith et al., 2016). B cells are detected in small numbers in the retinas of animals with EAU and their numbers increase late in disease (Boldison et al., 2014). In EAU, as in other autoimmune diseases, it has recently been established that IL-10 producing and IL-35 responsive B cells can modulate the progression of disease (Shen et al., 2014; Wang et al., 2014), but a role for B cells within the tissue remains unclear.

The limited data on the immune cell infiltrate – and the development of tissue niches - in human uveitic eyes is due to the lower incidence of uveitis compared to other autoimmune disorders (e.g. rheumatoid arthritis), and the technical difficulties of chorioretinal biopsy. Only small amounts of uveitic ocular tissue have been available for study. B cells are certainly a component of human disease: an immunohistochemical study of a single enucleated eye from a patient with juvenile idiopathic arthritis-associated uveitis demonstrated focal aggregates of CD20^+^ cells in the ciliary body (Parikh et al., 2008).

This report is now complemented by further data (Epps et al., manuscript in preparation), which characterises the immune cell infiltrate in 15 human eyes obtained from individuals with severe non-infectious uveitis (Anterior 1; Intermediate 1; Posterior 2; Panuveitis 11). B cells and T cells were detected in the majority (11 out of 15 cases [73%]) of cases (Fig. 2A). In 3 out 15 eyes (20%), there were clear features of ectopic lymphoid-like structures (ELS) (Fig. 2B). It was possible to identify focal aggregations of CD20^+^ B cells and CD3^+^ T cells (consisting of a mixed CD4^+^ and CD8^+^ population) with the segregation of lymphocytes into adjacent but distinct T and B cell zones (Fig. 2). CD21^+^ follicular dendritic cell (FDC) networks were present and there was evidence of the germinal centre reaction, (B cell lymphoma 6 protein (BCL6) and/or activation-induced cytidine deaminase (AID) expression within the CD20^+^ areas). Within a single eye, ELS of different degrees of maturity were observed, consistent with the local regulation of individual structures, rather than global co-ordination, and supporting the view that ELS are dynamic structures which develop and dissipate during the course of a chronic disease process, which has also been reported in rheumatoid arthritis (van de Sande et al., 2011). The fact that only a proportion of patients in this series – as in other studies of non-ocular tissues affected by chronic inflammation - developed features of ELS, may reflect underlying differences in the local inflammatory microenvironment predisposing towards or acting against the development of ELS (Jones et al., 2016). The small size of the series and limited access to other relevant information, such as HLA type, preclude more extensive mechanistic conclusions. The ocular structures that are involved extend beyond the retina, which is often the focus of analysis in acute EAU, and this may reflect important differences between chronic human disease and the more limited nature of most studies using EAU as a model.

The small numbers of lymphocytes found within the retina in human disease contrasts with the data of Kielczewski et al. (2016) describing ELS lesions in an EAU model driven by an antigen specific T cell receptor (TCR) transgenic mouse (the R161H mouse (Kielczewski et al., 2016)). In this model ELS is predominantly in the retina. Because TCR transgenesis leads to large numbers of T cells with specificity for a single retinal antigen, whereas disease following immunisation or in human uveitis is likely driven by a more diverse T cell population, such differences may impact the location of ELS. The anatomy of the choroid also differs between mouse and human: the choroid is relatively much thicker in the human eye and might provide a more permissive stromal microenvironment for the development of ELS. A caveat though is that the choroid is not well characterised in murine EAU.

The finding of ELS in the choroid in human uveitis has parallels with multiple sclerosis (MS), where ELS have been demonstrated within the dura mater of the meninges not the neural brain parenchyma (Magliozzi et al., 2007). Like the dura, the choroid is a highly vascularized structure containing abundant stromal fibroblasts which play a pivotal role in orchestrating the development of ELS (Buckley et al., 2015). Both structures are adjacent to a source of autoantigens (i.e. the neural retina and neural brain parenchyma), although it should also be borne in mind that the retina was severely damaged by the disease process in many of these samples and little retinal tissue remained.

Stromal cells in the choroid (such as fibroblastic reticular cells and blood endothelial cells) could play an important role in the recruitment and retention of immune cells. In chronic CNS inflammation, meningeal stromal cells upregulate secretion of the lymphoid chemokines, inflammatory cytokines and adhesion molecules (e.g. CXCL13; lymphotoxin, IL-17, IL-22; ICAM-1), mediating the recruitment and retention of immune cells into the CNS (Buckley et al., 2015; Columba-Cabezas et al., 2006; Pikor et al., 2015). In a subset of cases of chronic neuroinflammation, this leads to the formation of an immune-competent niche with structural compartmentalisation and the development of ELS (Choi et al., 2012; Gardner et al., 2013; Howell et al., 2011; Magliozzi et al., 2007, 2010).

In MS and its experimental model experimental autoimmune encephalomyelitis (EAE), immunoglobulin class-switched (and therefore antigen-experienced) B cell accumulations occur surrounding CD21^+^ CD35^+^ CXCL13^+^ stromal cells, a phenotype resembling FDCs (Magliozzi et al., 2007; Peters et al., 2011). The role of FDCs in lymphoid tissue in capturing antigen and presenting it to B cells to generate a germinal centre response is well-recognised (Heesters et al., 2014), and it can be surmised that these FDC-like stromal cells within the meninges perform the same function, resulting in the production of mature autoreactive B cells (memory B or plasma cells) in the target tissue, distant from central immune tolerance mechanisms, thus potentially exacerbating and perpetuating the chronic disease process (Magliozzi et al., 2007; Peters et al., 2011). In MS, a gradient of neuronal loss and demyelination is seen within areas of the cerebral cortex adjacent to sites of meningeal ELS formation, with more profound abnormalities occurring immediately proximal to the ELS and less marked abnormalities distally (Magliozzi et al., 2010). It is plausible that a similar scenario occurs in uveitis, with organised lymphoid tissue-like niches developing within the choroid adjacent to the neural retina, and thereby contributing to retinal pathology through the recruitment and retention of leukocytes.

B cells and stromal cells are not the only players in the formation of ELS. It is evident that Th17 cells play a pivotal role in the formation of ELS in EAE (Peters et al., 2011). Human meningeal post-mortem samples obtained from patients with a history of MS demonstrate an increased prevalence of RORγt^+^ lymphocytes in meningeal ELS compared to meninges displaying a diffuse inflammatory infiltration, implicating Th17 and/or group 3 innate lymphoid cells (ILS) in ELS formation (Serafini et al., 2016). Furthermore, in the EAE model ELS formation is not restricted to inflammation characterised by T cells of the Th17 compartment; adoptive transfer of Th1 or Th17-skewed cells resulted in the development of comparable levels of ELS formation (Pikor et al., 2015). Similarly, a CD4^+^ and CD8^+^ T cell infiltrate in addition to CD68^+^ macrophages and CD138^+^ plasma cells were evident in the data of Epps et al. from human uveitis.

The significance of ELS-like structures within the eye with respect to regulation or autoimmunity remains to be determined. Kielczewski's work on EAU (2016) correlates ELS with retinal function, with affected eyes having lower clinical signs of retinal inflammation initially as well as slower disease progression, as measured by electroretinography (Kielczewski et al., 2016), but losing this advantage as disease progresses. This contrasts with the majority of data on ELS in human autoimmunity, where ELS are associated with, for example, the generation of autoantibodies (Humby et al., 2009), a poorer clinical response to anti-TNF therapy in rheumatoid arthritis (Canete et al., 2009) and a worse clinical prognosis in secondary progressive multiple sclerosis (Magliozzi et al., 2007). This certainly raises the possibility that the presence of ELS in human uveitis may be associated with a worse clinical prognosis. There is emerging data that anti-B cell therapies may ameliorate human uveitis (Lasave et al., 2017), which is consistent with the B cell microenvironment of the ELS playing a pathogenic role in uveitis.

Further mechanistic data from experimental models is required to definitively establish the function of ELS within the ocular microenvironment in the context of chronic inflammation. In conjunction with such data, methods of establishing the presence of ELS and the dynamics of their development within the eye *in vivo* during uveitis are required: sampling ocular fluids for gene and protein expression patterns consistent with development of ELS and *in vivo* imaging exploiting enhanced depth imaging of the choroid and anterior segment imaging by optical coherence tomography (OCT) and potentially *in vivo* labelling of lymphocytes opens this possibility. Thus, the evidence of tissue re-programming seen in chronic immune activation within the human eye – both in the form of immune cell spatial reorganisation into B cell follicle-like structures and vascular re-programming - results in the formation of a potential niche for antigen-specific B cell proliferation, mutation, selection and maturation, B-T cell interactions, and could be of clinical relevance with respect to B-cell immunomodulatory therapies in uveitis.

### 3.4 Neutrophils

Although neutrophils are best known as acute pro-inflammatory responders, they have also been implicated in tissue surveillance, responding to pertussis toxin-sensitive signals (Ng et al., 2011). Neutrophils identified by their expression of Ly-6G are not found in the normal retina. However, they can be detected early in the disease prodrome and in the B10.RIII uveitis model peak at the same time as CD4^+^ IL-17 expression (Kerr et al., 2008b). This pattern is not seen in C57BL/6 animals where Ly-6G positive cells continue to be detected at similar low levels (<1000 cells) through to late time points in the disease course (J. Boldison, unpublished data). Gr-1 positive cell recruitment is regulated by G-CSF via CXCR2 and CXCR4 and targeting this cytokine leads to a selective deficit in Gr-1 positive cells and reduced EAU (Goldberg et al., 2016). Genetic ablation of IFNγ exacerbated clinical EAU and was associated with a marked increase in the accumulation of granulocytes. There was an altered pattern of dominant chemokine expression (CCL1, 11, 17, 22 and CXCL2 in the IFNγ knockouts and CXCL9, 10, 11 and CCL5 in wild type disease) and granulocyte depletion ameliorated disease (Jones et al., 1997; Su et al., 2007).

### 3.5 Microglia and macrophages

Flow cytometry analysis of the normal retina identifies a population of fewer than 1000 CD11b^+^ cells (Table 1). These cells are resident microglia (Hughes et al., 2003; Liu et al., 2013). In healthy tissue, microglia form ramified networks in which individual cells have a limited location within which they move to survey the environment (Luckoff et al., 2017; McMenamin, 1999). In models of several different ocular disease, these cells relocate. As indicated above, following injury, infection and danger signals, they move to the subretinal space and some assume a more amoeboid form that becomes a common component of the recruited cell population (Chinnery et al., 2015; Ng and Streilein, 2001; Zinkernagel et al., 2013). Microglial accumulation in the subretinal space (Karlstetter et al., 2010) is also a feature of the aging retina, reinforcing that this process is part of a general response mechanism to stress. Relocation is regulated by cytokines, chemokines and signalling receptors (Carter and Dick, 2004; Luhmann et al., 2012) that direct cell migration, and this occurs in human explants as well as murine systems. The expression of other molecules may also be important in this process and future research is likely to reveal other important mechanisms.

Monocyte/macrophage activation is controlled by both positive and negative signals from the local environment. One important receptor that delivers a negative signal to these cells is the CD200R, which plays a role in modulating inflammation under a wide range of pathologies (Gorczynski et al., 2010; Jenmalm et al., 2006; Snelgrove et al., 2008). The ligand for CD200R is widely expressed and has also been co-opted by a number of different viruses to which it presumably imparts an advantage (Barclay et al., 2002). In the eye the role of CD200/CD200R in the control of the local environment extends beyond just regulation of inflammation (Broderick et al., 2002; Copland et al., 2007) to the control of other functions such as angiogenesis (Horie et al., 2013).

In EAU in C57BL/6 mice, myelomonocytic cells, visualised with various fluorescent reporters, accumulate around the optic nerve head and along retinal vessels. They are clearly present by day 14 post-immunisation (Chen et al., 2015). As uveitis develops, there is an increasing contribution of CD11b^+^ cells recruited from the circulation; their ablation can prevent the development of clinical disease, an effect that is partly mediated by the reduction in NO (Forrester et al., 1998; Hoey et al., 1997). The phenotype of macrophages in the circulation differs significantly from those that accumulate in the retina which upregulate Gr-1 (Kerr et al., 2008b). The recirculation of these monocytes is inhibited by blocking CD44 or CD62L (Xu et al., 2008) and unlike CD4^+^ T cells their recruitment is conditioned by activation through the TNFR1, because in mixed chimeras of TNFR1 expressing and non-expressing macrophages, only the cells that express TNFR1 accumulate in the retina (Raveney et al., 2009). On the other hand, their expression of CCR2 is dispensable to the development of disease (Dagkalis et al., 2009). When monocyte/macrophages are purified from the eye at the peak of disease, they are found to support antigen specific activation of cytokine secretion by T cells, but they restrict T cell proliferation which is inhibited through NO and PGE_2_ dependent mechanisms. This serves to produce a microenvironment in which energy that would otherwise be used for T cell division, is available to other effector cells such as activated macrophages and neutrophils (Raveney et al., 2010). There are other circumstances in which T cells can proliferate within the ocular environment. Naive transgenic T cells, that recognised an ocular autoantigen, and were transferred directly into the eye both proliferated and expressed a FoxP3 associated GFP-reporter, indicating that they were of a regulatory phenotype; a similar transfer into animals with established uveitis did not lead to conversion to a Treg phenotype and their degree of proliferation was not reported (Zhou et al., 2012). How changes in the capacity to support local proliferation vary with time and changes in the local environment awaits further clarification.

### 3.6 Angiogenesis

Inappropriate angiogenesis is a source of many different types of ocular pathology. It is regulated by a large number of soluble factor such as the VEGF family (Nagy et al., 2007), Lrg1 (Wang et al., 2013), thrombospondin-1 (Dipietro and Polverini, 1993) and also receptor mediated interactions with the chemokine ligands and the extracellular matrix (Campochiaro, 2015; Chen et al., 2012). In EAU, angiogenic effectors such as VEGF can disrupt the blood-retinal barrier (Luna et al., 1997) and although angiogenesis is not a prominent component of human inflammatory eye disease, vascular dysfunction leading to macular oedema is a common late sight threatening complication (Daruich et al., 2017; Rothova et al., 1996). Such persistent ocular inflammation is associated with neovascular lesions in a number of different manifestations of human ocular disease, including choroidal neovascularisation and retinal angiomatous proliferation (Dhingra et al., 2010; Kedhar et al., 2007; Thorne et al., 2006).

**Fig. 3:**
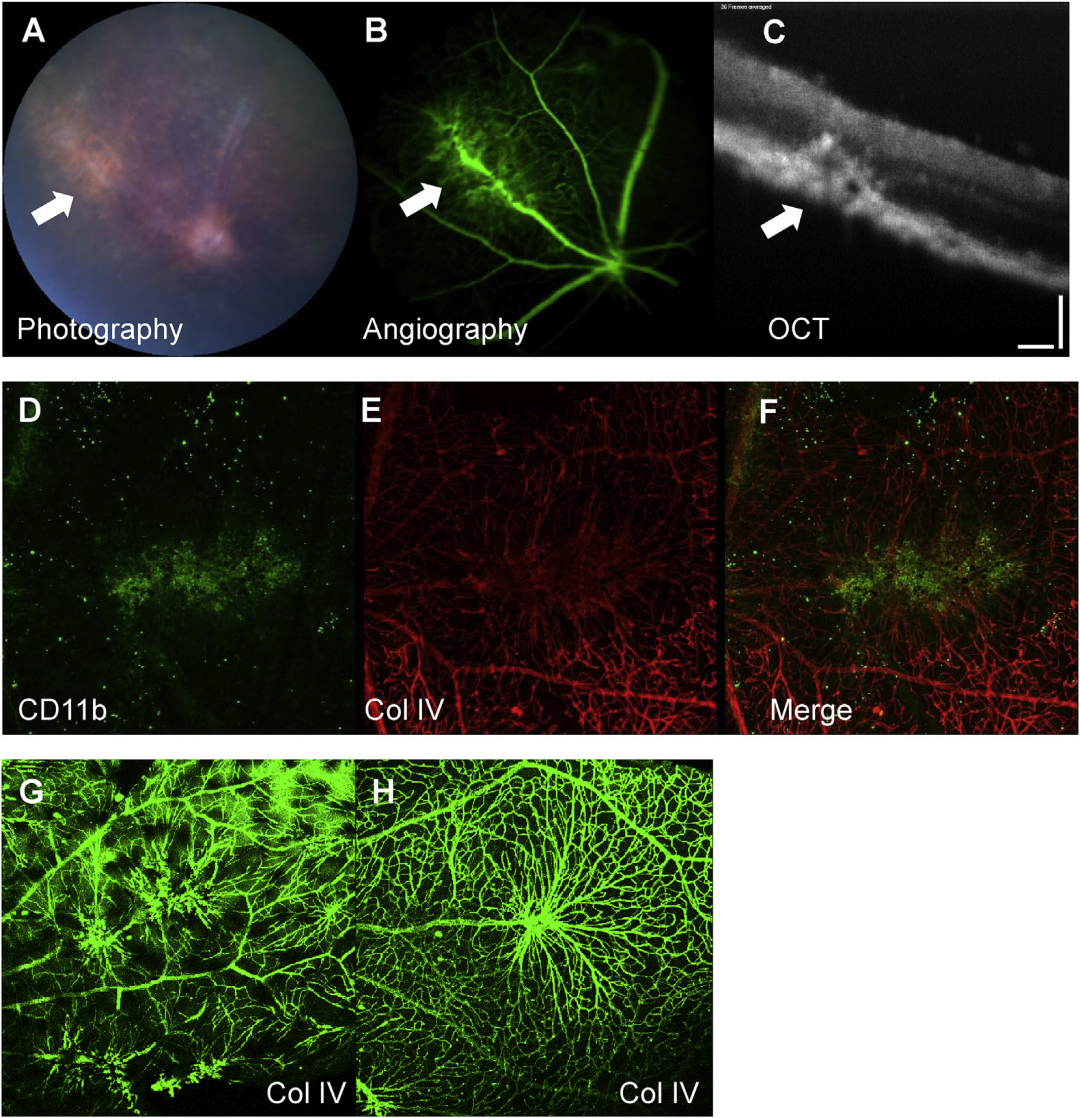
Animals (C57BL/6) were immunized with IRBP 1–20 to induce EAU and clinical disease was monitored by topical endoscopic fundal imaging (TEFI), fluorescein angiography and optical coherence tomography. At day 146 persistent disease neovascular membranes (white arrows), extending into the retina and fused with the RPE are seen (A–C). The neovascular membranes in wild type animals are associated with CD11b positive macrophages (D–F) and disruption of collagen IV. Gene manipulations that target macrophage function can modify the vascular phenotype, for example when C57BL/6 wild type (G) and C57BL/6 TNFR1 (H) knockout animals with persistent EAU were assessed 90 days after the induction of EAU by preparing retinal flatmounts and staining with collagen IV, confocal microscopy showed more vascular modifications in the wild type animals. Photography, angiography and OCT were carried out using TEFI and a micron IV retinal imaging microscope (Phoenix research laboratories). Collagen IV stain used a primary rabbit anti-mouse antibody and secondary staining with goat anti-rabbit conjugated with AlexaFluor 568 (red E,F) or AlexaFluor 488 (green G,H). Scale bar panel C 100 microns.

In C57BL/6 mice a similar phenotype occurs in the course of EAU (progressing from around day 60) manifesting as the formation of retinal neovascular membranes (RNM) that extend through the retina and fuse with the retinal pigment epithelium (RPE) (Chen et al., 2012). This phenotype has been visualised by photography, OCT and fluorescein angiography as late as 146 days after the induction of disease (Fig. 3A–C). At this time, Th17 cell (not shown) and macrophage (CD11b^+^) infiltration is found associated with the lesions (Fig. 3D–F). Manipulations that modify the accumulation of leukocytes (knockouts of CCR2, CCL2xCX_3_CR1 and TNFR1) or that modify the extracellular matrix (Thrombospondin-1 knockout) change the severity of RNM angiogenesis (Chen et al., 2012; Fordham et al., 2012; Raveney et al., 2009; Zhao et al., 2014) (T.Khera, M.Stimpson et al. unpublished). This demonstrates that the nature of the local microenvironment informs the development of the new vessels.

Because expression of TNFR1 is critical for the efficient recruitment of macrophages in EAU (Raveney et al., 2009), we compared angiogenesis in persistent uveitis in TNFR1 knockout and C57BL/6 WT animals 90 days after the induction of disease, and found that there was a striking difference in the phenotype of the vascular changes (Fig. 3G and H). At this time point, the retinas of both strains are heavily infiltrated with leukocytes, compared to normal age-matched animals. Total cell numbers are lower in the knockout mice, but the ratio of CD4^+^:CD11b^+^ cells is the same in both strains. When the cytokine content of the retinas was assessed by measuring RNA, IL-17 but not IFNγ levels were decreased in the TNFR1 knockouts (T.Khera, M.Stimpson et al. unpublished). This shows that different patterns of cytokine signalling, that impact on the phenotype of recruited macrophages, can modulate the nature of the local microenvironment and influence the development of new vessels. Such macrophages remain a tractable target for therapy (Lee et al., 2014).

One cellular feature of this persistent ocular inflammation, the ongoing presence of macrophages exhibiting an alternative activation phenotype (Lee et al., 2014) has been implicated in several different aspects of tissue remodelling including angiogenesis. Inflammatory and anti-inflammatory cytokines and/or the uptake of damaged RPE can condition the macrophage to adopt a phenotype that can promote or limit new vessel growth (Liu et al., 2013; Wu et al., 2010). Acute conditioning *in vitro* and in *in vivo* with the cytokine IL-4 leads to a macrophage phenotype that inhibits angiogenesis. This occurs through the upregulation of a decoy receptor that can neutralise vascular endothelial growth factor (VEGF), sufficient to suppress the development of laser-induced choroidal neovascularisation (Wu et al., 2015). This work delivers proof of principle for macrophage targeting to modulate angiogenesis.

## 4 Summary and future directions

It seems reasonable to propose that the process of local tissue inflammation, within an immune-privileged tissue, has re-programmed immunosurveillance and induced new niches that become occupied by cells of the immune system. Such a scenario need not imply the differentiation of immune cells within the eye, but rather the development of sites where such cells halt, and where they have an increased dwell time much greater than that seen in the naive state. These sites might have a defined anatomical distribution, perhaps associated with the perivascular space, or their distribution could relate to sites of previous inflammation as seen in CNS tissue (Wakim et al., 2010). The use of FTY-720, a drug that traps recirculating lymphocytes, to inhibit lymphocyte recirculation six weeks after the induction of disease in C57BL/6 mice, revealed striking differences in the tissue half-life of CD8^+^ T cells compared with CD4^+^ and CD11b^+^ cells (Boldison et al., 2014) and begins to address these issues of differential trafficking and cell dynamics. Theoretical studies also support the importance of the kinetics of cell trafficking into regions outside the conventional secondary lymphoid compartment (Nicholson et al., 2012) as a key factor determining the progress of the disease process. Finally, such niches could offer attractive therapeutic targets. If local immune surveillance can be restored to its pre-disease state, local triggering and relapse might be limited and ongoing tissue damage impeded.

Our future understanding of immune privileged tissue re-programming in uveitis will be facilitated by new technologies which are advancing rapidly. Tackling the difficult issues of the local organisation of transient cellular structures will become possible as techniques such as OCT achieve resolution at the level of individual cells (Drexler et al., 2014). Complementary studies that can recover and analyse the transcriptome of single cells, from within the tissue microenvironment (Singer et al., 2016; Stubbington et al., 2016), will allow us to move beyond low dimensional analysis based on the expression of only one or two reporter molecules to whole cell physiology. By examining the heterogeneity of cells that make up different niches we can begin to understand how these dynamic structures are formed, maintained and dissolved. One trend, that is already presaged by changes in monocyte phenotype on relocation from the circulation to the tissue (Kerr et al., 2008b), is the integration of cell surface phenotype with reprogramming lipid biosynthesis (Wang et al., 2015). The key idea of a dialogue between the tissue and the immune response will be an increasingly fertile area for research, as we are better able to discern both sides of the conversation.
